# Rhizosphere analysis of field-grown *Panax ginseng* with different degrees of red skin provides the basis for preventing red skin syndrome

**DOI:** 10.1186/s12866-021-02430-9

**Published:** 2022-01-06

**Authors:** Ling Dong, Xingbo Bian, Yan Zhao, He Yang, Yonghua Xu, Yongzhong Han, Lianxue Zhang

**Affiliations:** 1 National& Local Joint Engineering Research Center for Ginseng Breeding and Development, Jilin Agricultural University, Changchun, China; 2 College of Chinese Medicinal Materials, Jilin Agricultural University, 130118 Changchun, Jilin Province China; 3 Jilin Provincial Ginseng and Pilose Antler Office, Changchun, China

**Keywords:** Ginseng red skin root syndrome, rhizosphere, Soil ecological environment, Microbial interaction network

## Abstract

**Background:**

Ginseng red skin root syndrome (GRS) is one of the most common ginseng (*Panax ginseng* Meyer) diseases. It leads to a severe decline in *P. ginseng* quality and seriously affects the *P. ginseng* industry in China. However, as a root disease, the characteristics of the GRS rhizosphere microbiome are still unclear.

**Methods:**

The amplicon bacterial 16 S rRNA genes and fungal ITS (Internal Transcribed Spacer) regions Illumina sequencing technology, combined with microbial diversity and composition analysis based on R software, was used to explore the relationship between soil ecological environment and GRS.

**Results:**

There were significant differences in the diversity and richness of soil microorganisms between the rhizosphere with different degrees of disease, especially between healthy *P. ginseng* (HG) and heavily diseased groups. The variation characteristics of microbial abundance in different taxa levels were analyzed. The interaction network of rhizosphere microorganisms of *P. ginseng* under GRS background was established. We also found that different *P. ginseng* rhizosphere microbial communities have multiple changes in stability and complexity through the established interaction network. Microbes closely related to potential pathogenic fungi were also identified according to the interaction network, which provided clues for looking for biological control agents. Finally, the Distance-based redundancy analysis (dbRDA) results indicated that total phosphorus (TP), available potassium (AK), available phosphorus (AP), catalase (CAT), invertase (INV) are the key factors that influence the microbial communities. Moreover, the content of these key factors in the rhizosphere was negatively correlated with disease degrees.

**Conclusions:**

In this study, we comprehensively analyzed the rhizosphere characteristics of *P. ginseng* with different levels of disease, and explored the interaction relationship among microorganisms. These results provide a basis for soil improvement and biological control of field-grown in the future.

**Supplementary Information:**

The online version contains supplementary material available at 10.1186/s12866-021-02430-9.

## Introduction

Ginseng (*Panax ginseng* C, A, Mayer), mainly distributed in northeast China and South Korea, is known as the “King of Herbs” because of its substantial medicinal value. Because of *P. ginseng*’s continuous cropping effect, the deforestation planted *P. ginseng* pattern was often used in China before. For a long time, we have lost many forest resources for the *P. ginseng* cultivation. Moreover, now, this pattern of cultivation is no longer allowed. Due to the limitations of planting areas and planting patterns, it is more critical to improving *P. ginseng*’s the quality and yield for the sustainable development of the *P. ginseng* industry. Besides, *P. ginseng* as a perennial plant needs several years of cultivation, and its cultivation has risk against several kinds of stresses. Many diseases that occur during *P. ginseng*’s growth process are the biggest obstacles to obtaining high yield and quality *P. ginseng*.

GRS is characterized by rust-stained reddish-brown areas on the epidermis of *P. ginseng* roots. Areas may grow more significant as the planting years increase. In severe cases, areas can occupy more than 80% of the epidermis of *P. ginseng* roots. Unlike root rot disease, severe red skin roots are also rarely showing root rot [1]. Previous studies have found that the chitosan application induces red skin roots, and a variety of phenolic compounds and elements accumulate in red skin tissues [2]. In particular, the accumulation of Al and Fe ions may promote the accumulation of phenolic compounds and the activation of enzymes related to their oxidation. Various antioxidant substances and antioxidant enzymes in red skin tissues are significantly increased, preventing phenolic compounds from being oxidized [3]. The microorganisms produce pectinase, cellulase, and ligninase that damage the cell walls of *P. ginseng* roots, causing red skin symptoms exacerbated by the application of Fe^3+^ [4]. *Ilyonectria* is a pathogenic fungus with high pathogenicity to *P. ginseng*. The isolates of *Ilyonectria* species that infect *P. ginseng* are divided into four species: *I. robusta*, *I. mors-panacis*, *I. panacis*, and *I. crassa*. It has been reported that *Ilyonectria* may be the pathogenic fungus of GRS, and abiotic factors seem to be closely related to the formation of red skin [1, 5–9].

The results of previous studies showed that the rhizosphere of diseased *P. ginseng* was separated from that of healthy *P. ginseng*, and there are considerable differences in the dominant bacteria and fungi genera in the two rhizospheres. Some beneficial microorganisms were significantly reduced in the rhizosphere of diseased *P. ginseng* [6]. Moreover, the diversity of bacteria and fungi in the rhizosphere of diseased *P. ginseng* was decreased [10]. There have also been studies exploring rhizospheres of plants with varying degrees of red skin and have found a strong correlation between metal element accumulation and red skin symptoms. According to the experimental results, *Ilyonectria* may not be necessary for red skin [11].

There are close interactions between plants, soil, and microorganisms [12]. Healthy plant growth is dependent on the ability of microorganisms to promote soil material circulation and nutrient conversion [13, 14]. The plants’ root diseases are closely related to soil microbial community and physical and chemical properties, and there are a variety of potential pathogens in soil [15]. Similarly, various factors affect rhizosphere microorganisms, such as changes in root exudates, differences in soil characteristics associated with root diseases, and changes in root morphology of diseased plants [16]. It can be seen that the occurrence of GRS is probably closely related to the rhizosphere characteristics. The rhizosphere of *P. ginseng* is likely to change with the occurrence of GRS. Therefore, to study the GRS, it is necessary to understand the rhizosphere’s microbial community and physical and chemical properties.

In this study, we classified the *P. ginseng* rhizosphere into five degrees based on the severity of the disease. The 16 S rRNA gene and ITS region were applied to explore the five degrees rhizosphere microbial community composition on the Illumina PE250 platform. Nutrient composition and enzyme activity in the rhizosphere were also examined, and their relationship with microbial changes was explored. This study elucidated the evolution rule of *P. ginseng* rhizosphere with the aggravation of GRS and provides the basis for preventing and treating this disease in field production.

## Results

### Richness and diversity of microbial communities

In the bacterial analysis based on 16 S rRNA, the operational taxonomic units (OTUs) number in the GRS2 group was significantly higher than that in the HG group, GRS1 group, and GRS3 group (P < 0.05). The Observed species of the GRS2 group is also substantially higher than HG and GRS3 (P < 0.05). Besides, The Shannon index and Simpson index of the GRS2 group are both significantly higher than that of other groups, and the Chao1 index of the GRS2 group is also significantly higher than that of the HG group and GRS3 group (P < 0.05) (Table 1).

**Table 1:** Diversity of the 16 S rRNA gene-based bacterial and ITS rRNA gene-based fungi communities. Values are the means ± standard errors (n = 6). Different letters in the same row mean significant difference at P <0.05 among the five treatments. HG (healthy ginseng), GRS1 (rust root area greater than 0, less than or equal to 25%), GRS2 (red skin root area greater than 25%, less than or equal to 50%), GRS3 (rust root area greater than 50%, less than or equal to 75%) and GRS4 (rust root area greater than 75%)

	OTUs	Observed species	Shannon	Simpson	Chao1
**Bacteria**					
HG	2209 ± 163 b	1952 ± 125 b	8.518 ± 0.078 bc	0.993 ± 0.001 b	2171.36 ± 140.73 bc
GRS1	2226 ± 183 b	2013 ± 111 ab	8.604 ± 0.116 bc	0.993 ± 0.001 b	2400.47 ± 365.58 ab
GRS2	2510 ± 198 a	2225 ± 156 a	8.849 ± 0.073 a	0.994 ± 0.000 a	2475.05 ± 166.26 a
GRS3	2124 ± 191 b	1874 ± 168 b	8.369 ± 0.219 c	0.991 ± 0.002 b	2096.48 ± 182.25 c
GRS4	2297 ± 250 ab	2035 ± 219 ab	8.606 ± 0.254 b	0.992 ± 0.002 b	2278.28 ± 245.57 abc
**Fungi**					
HG	593 ± 103 a	533 ± 85 ab	4.822 ± 0.111 a	0.913 ± 0.005 a	576.98 ± 102.37 ab
GRS1	602 ± 132 a	537 ± 114 ab	4.866 ± 0.243 a	0.927 ± 0.009 ab	595.66 ± 134.21 ab
GRS2	615 ± 84 a	568 ± 84 a	4.74 ± 0.209 a	0.914 ± 0.015 ab	717.49 ± 244.41 a
GRS3	489 ± 93 a	436 ± 75 b	4.348 ± 0.0.5 b	0.905 ± 0.008 b	486.28 ± 86.68 b
GRS4	546 ± 140 a	489 ± 120 ab	4.497 ± 0.263 b	0.897 ± 0.016 b	549.05 ± 139.95 ab

Based on the ITS rRNA analysis of fungi, there was no significant difference in OTUs numbers among the five groups. The Observed species and the Chao1 index of the GRS2 group were significantly higher than that of the GRS3 group (P < 0.05). Also, the Shannon indices of the HG, GRS1 and GRS2 groups were considerably higher those of the GRS3 and GRS4 groups (P < 0.05). The Simpson index was significantly higher in the HG group than in the GRS3 and GRS4 groups (P < 0.05) (Table 1).

## Differences and composition of microbial communities

**Fig. 1:**
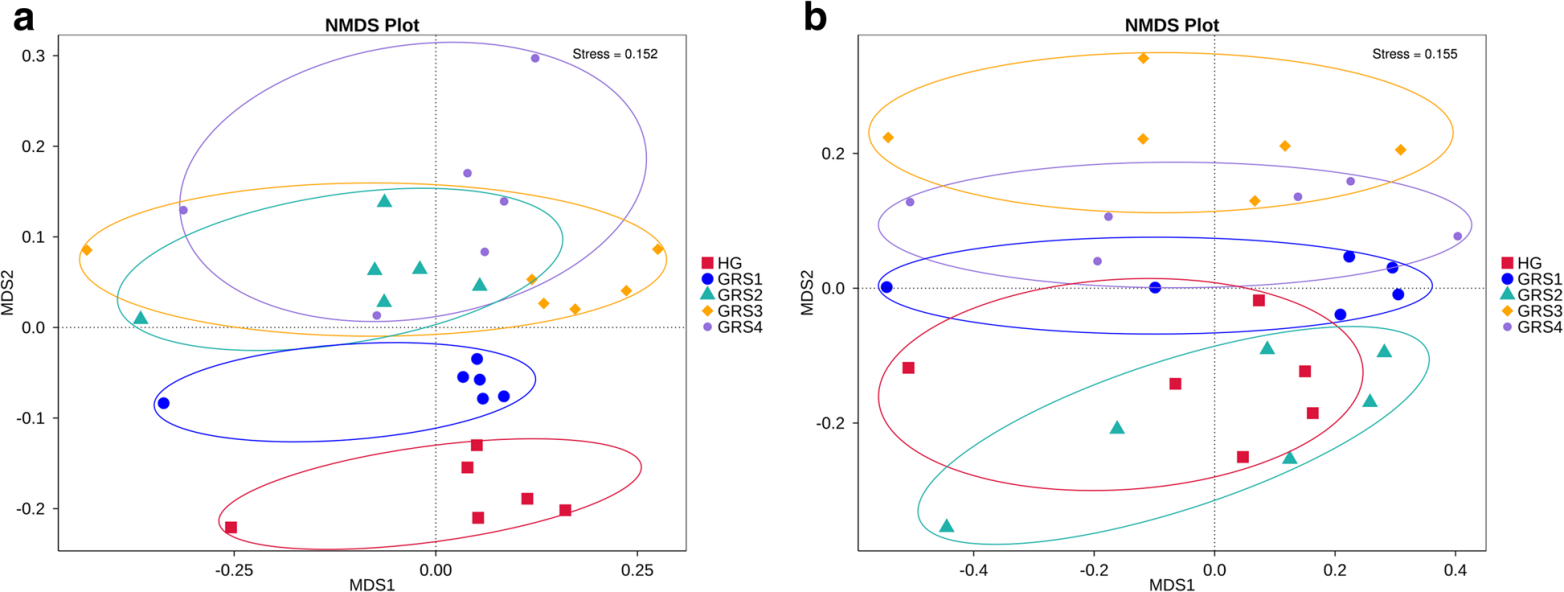
Non-Metric Multi-Dimensional Scaling (NMDS) analysis plot. **A** bacteria; **B** fungi. HG (healthy ginseng), GRS1 (rust root area greater than 0, less than or equal to 25%), GRS2 (red skin root area greater than 25%, less than or equal to 50%), GRS3 (rust root area greater than 50%, less than or equal to 75%) and GRS4 (rust root area greater than 75%)

**Fig. 2:**
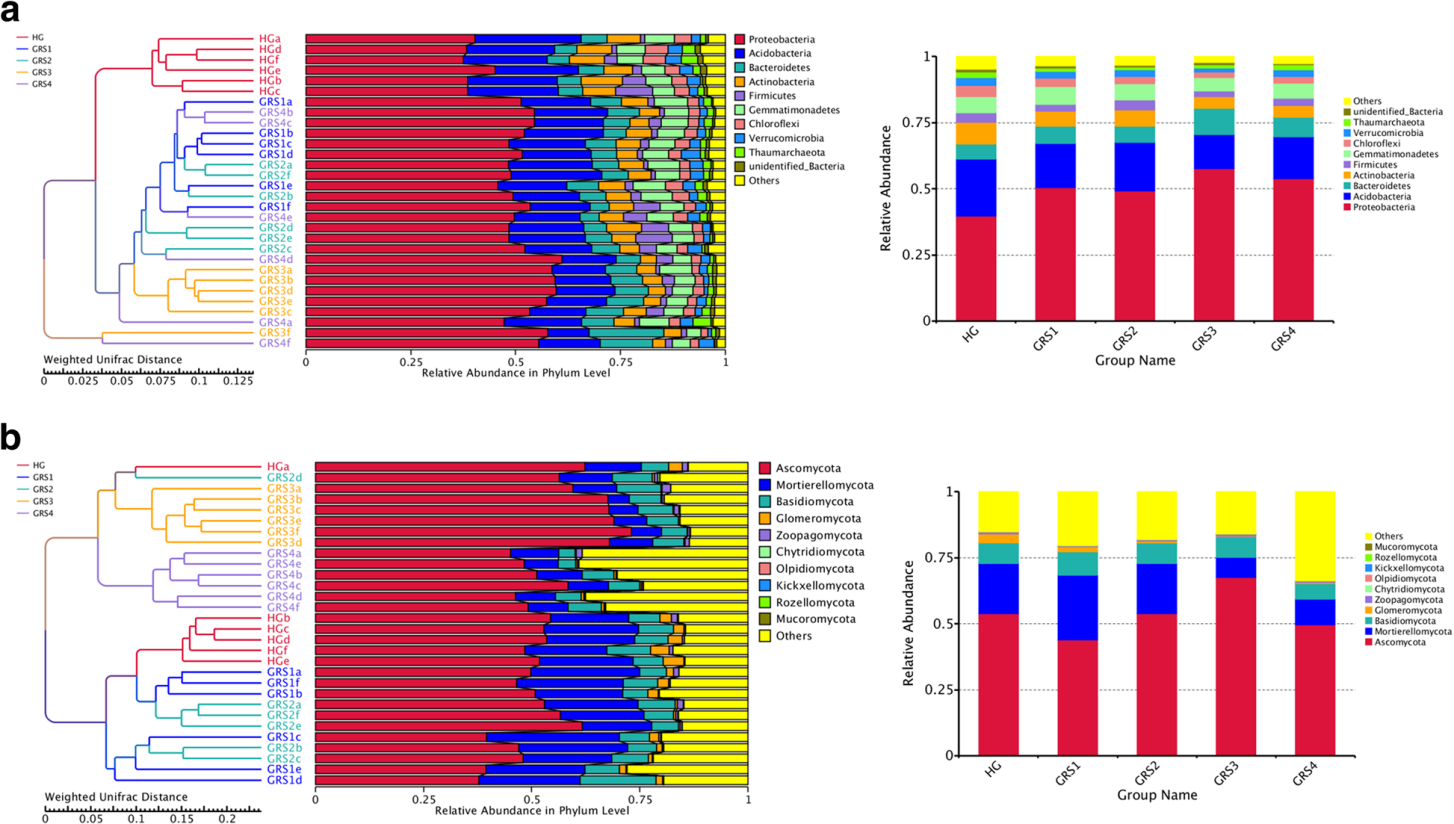
Unweighted Pair-group Method with Arithmetic Mean (UPGMA) clustering analysis with Weighted Unifrac distance matrix and the relative abundance of each sample and group at the phylum level. **A** bacteria; **B** fungi

In the results of the ANOSIM analysis, the inter-group differences were higher than the intra-group differences (R-value > 0), and the statistical analysis was significant (P-value < 0.05) except for GRS2-GRS4 (Table S3). The Non-Metric Multi-Dimensional Scaling (NMDS) analysis based on bacterial OTUs level showed no significant separation of GRS2, GRS3, and GRS4 groups in all five groups of samples. In contrast, HG and GRS1 groups were separated from other groups (Fig. 1 A). Cluster analysis showed that samples from the HG and GRS3 groups were significantly clustered to form independent clusters. The samples were then analysed for bacterial abundance, belonging to 46 phyla, 246 families, and 593 genera. In the analysis of phylum level, Proteobacteria is the most dominant phylum (average 50.2%), followed by Acidobacteria (average 17.6%), Bacteroidetes (average 7.1%) Gemmatimonadetes (average 6.0%), and Actinobacteria (average 5.9%) (Fig. 2 A).

In terms of the fungi community, the results of ANOSIM analysis showed that there were significant differences among all groups (R-value > 0, P-value < 0.05) (Table S3). By NMDS analysis, we found the significant separation of HG and GRS2 groups from GRS3 and GRS4 groups (Fig. 1B). Unweighted Pair-group Method with Arithmetic Means (UPGMA) cluster analysis showed that GRS3 and GRS4 samples were significantly clustered and formed independent clusters. In HG group, HGa did not cluster with other HG groups. Based on the ITS gene sequences annotation, we identified 12 phyla, 197 families, and 323 genera in the fungal community. At the phylum level, Ascomycota is the most dominant (average 53.8%), followed by Mortierellomycota (average 16.0%), Basidiomycota (average 7.6%), and Glomeromycota (average 1.3%) (Fig. 2B).

## Biomarkers and different levels of taxa change

**Fig. 3:**
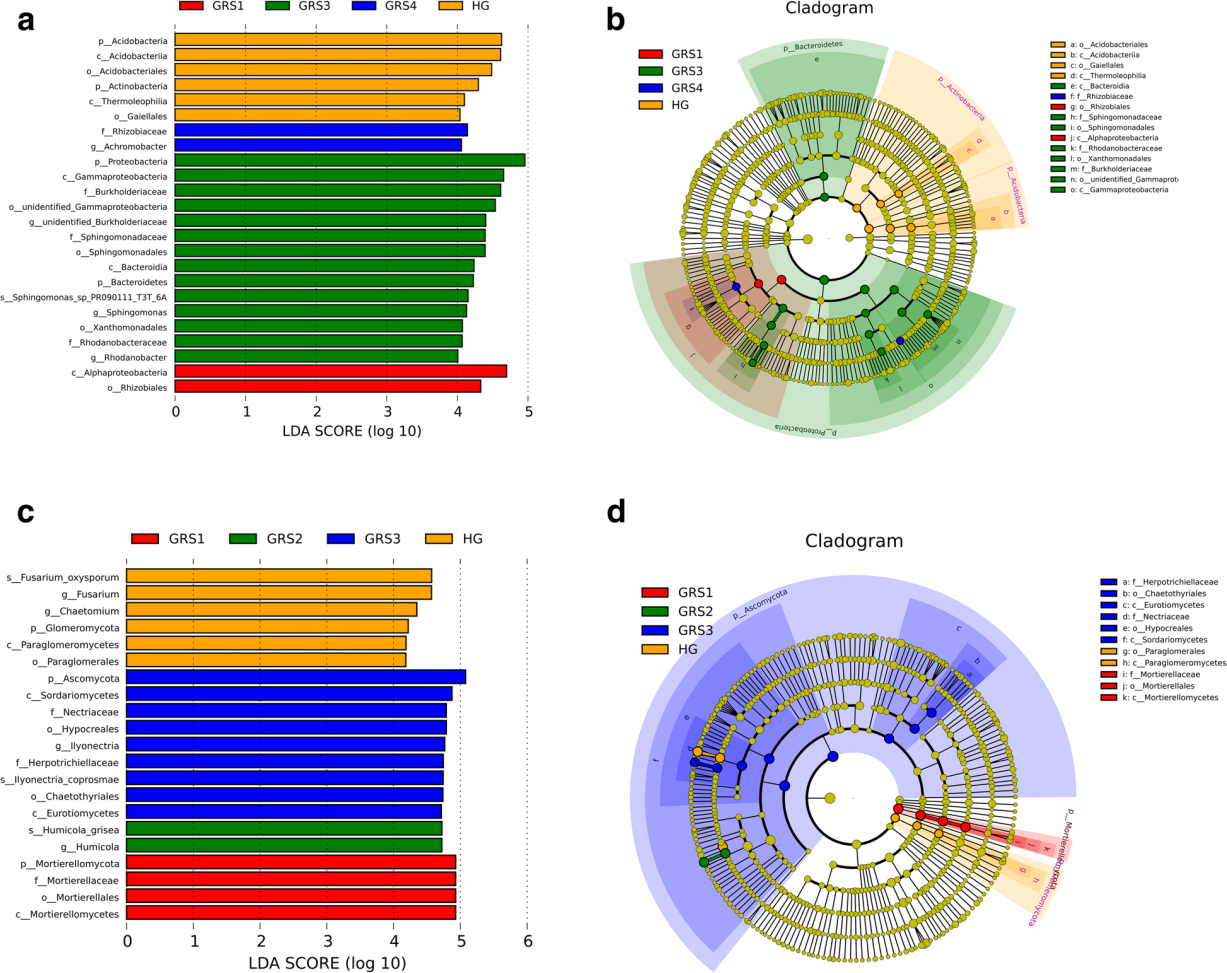
The linear discriminant analysis (LDA) effect size (LEfSe) analysis. **A** LDA scores of bacteria differential taxa (LDA score>4); **B** diagram of bacterial differential taxa; **C** LDA scores of fungi differential taxa (LDA score>4); **D** diagram of fungi differential taxa

Through linear discriminant analysis (LDA) effect size (LEfSe) analysis, we found six bacterial taxa in the HG group with LDA scores greater than 4, including Acidobacteria and Actinobacteria. In the GRS1 group, LDA scores greater than 4 were Alphaproteobacteria and Rhizobiales. Phyla level with LDA scores greater than 4 in GRS3 was Proteobacteria and Bacteroidetes. Potential biomarkers of the GRS4 group were selected as Rizobiaceae and *Achromobacter* (Fig. 3 A and Fig. 3B). Then, we conducted MetaStat analysis (from the phylum level to the species level) to understand further the bacterial community changes with the disease severity of *P. ginseng* (Fig. S2). The results showed that the abundance of specific bacterial taxa in the rhizosphere at different levels varied significantly with varying degrees of disease. Notably, at the phylum level, compared with the HG group, the abundance of Proteobacteria in the four red skin groups was significantly increased, and Bacteroidetes has a greater abundance in GRS3 and GRS4 groups. The abundance of Actinobacteria, Chloroflexi, and Gemmatimonadetes were lower in the severe groups (GRS3 and GRS4) than in the healthy and mild groups (GRS1 and GRS2). Interestingly, the genera with a relatively high abundance were concentrated in HG, GRS3, and GRS4 groups, and there were also significant differences between the two severe groups (GRS3 and GRS4).

LEfSe analysis results for fungi showed that, in the HG group, *Fusarium*, *Chaetomium*, and Glomeromycota had LDA scores over 4. In the GRS1 group, Mortierellomycota were screened out. And in GRS2 group, *Humicola* had LDA scores over 4. Besides, there were more taxa with LDA scores above 4 in the GRS3 group, and they were concentrated in Ascomycota, including Nectriaceae, Herpotrichiellaceae, and *Ilyonectria* (Fig. 3 C and Fig. 3D). For the MetaStat analysis at the phylum level, the results showed that the relative abundance of Mortierellomycota, Glomeromycota, and Mucoromycota decreased with increasing *P. ginseng* red skin area (Fig S3). Besides, among several fungi with greater relative abundance at the genus level, the GRS3 and GRS4 groups had a much greater abundance of *Ilyonectria* than the other three groups. In contrast, the abundance of *Trichosporon* and *Paraglomus* decreased with the increase of red skin area.

## Network analysis of microbial communities

**Fig. 4:**
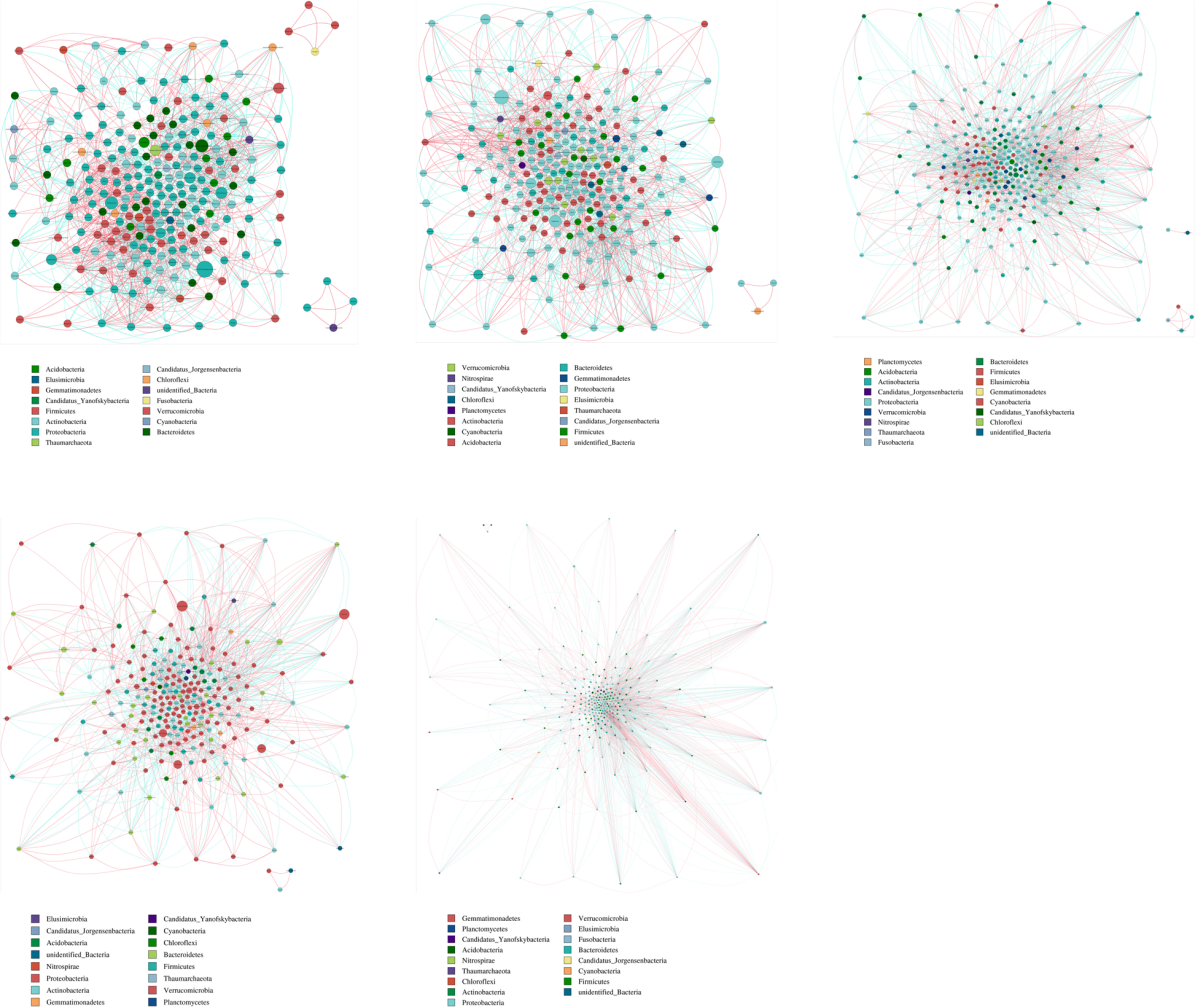
Overview of bacterial networks. Different nodes represent different genera; the size of nodes represents the average relative abundance of the genus; the nodes of the same gate have the same color; the color of the lines between nodes corresponds to the positive and negative correlation (red is positively correlated; blue is negatively correlated)

**Fig. 5:**
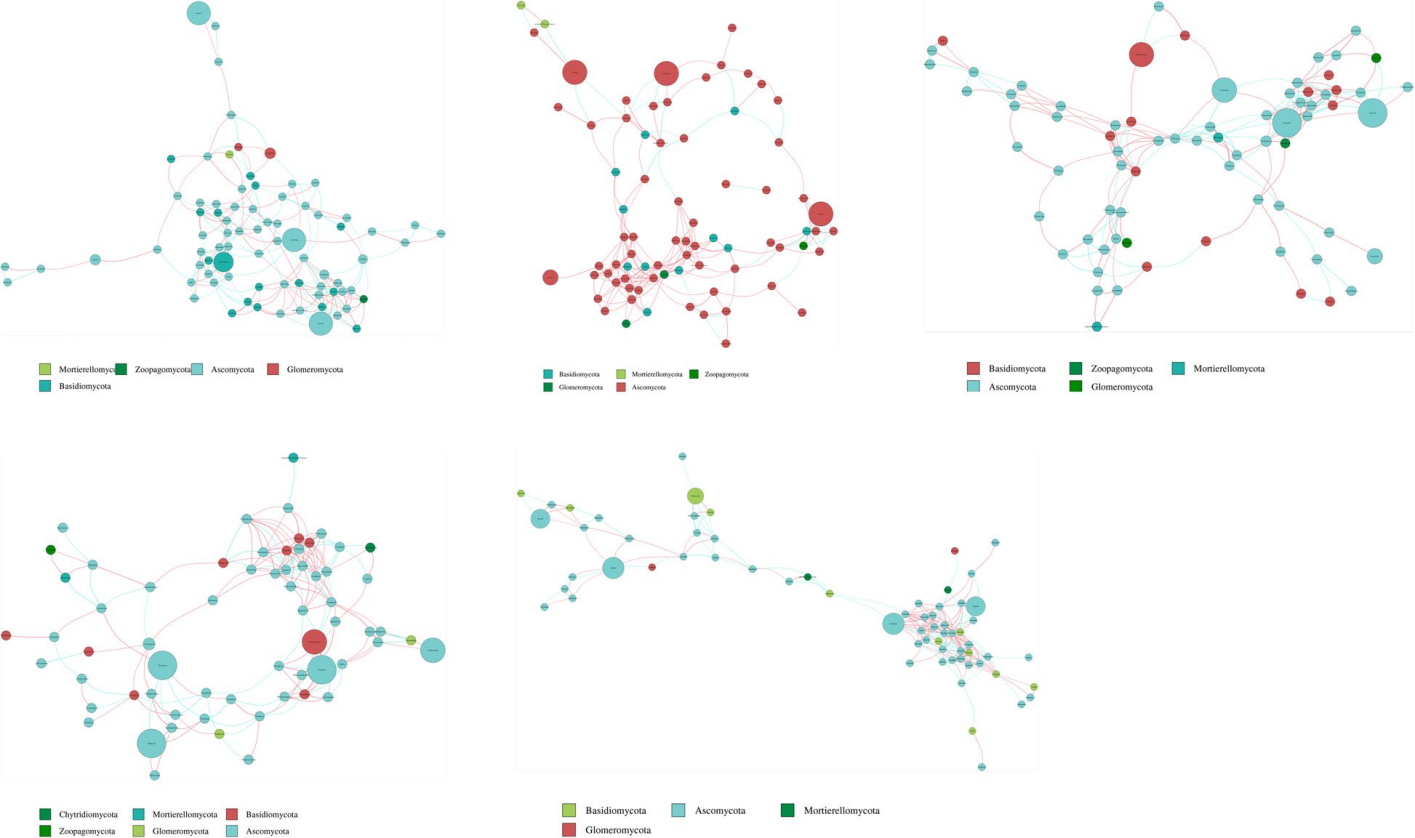
Overview of fungi networks. Different nodes represent different genera; the size of nodes represents the average relative abundance of the genus; the nodes of the same gate have the same color; the color of the lines between nodes corresponds to the positive and negative correlation (red is positively correlated; blue is negatively correlated)

Based on the Spearman correlation, microbial networks in five groups from HG to GRS4 were established. The clustering coefficient values, characteristic path length, and modularity of non-random networks were higher than the values for their corresponding random network, suggesting that interactions in the current networks were significantly different from randomly generated networks (Table S4). The highest connectivity phylum in a network is often considered as the keystone taxa [17]. Through microbial networks, we can find that the keystone phyla of bacteria and fungi are Proteobacteria and Ascomycota, respectively (Figs. 4 and 5). In the bacterial network, the number of nodes in the five groups did not change significantly. However, the number of total links in the five groups was not stable, reaching the maximum in the GRS4 group (3600). All four red skin groups increased their proportion of negative links compared to the HG group (Table S4). Among other topological properties, the average path length of the GRS4 group was lower than that of other groups. The modularity of red skin groups was more down than the HG group, especially the GRS4 group. The GRS4 group clustering coefficient and average degree were higher than the other groups (Table S4).

For fungi, the GRS4 group had significantly more links than the other groups, and the four red skin groups had more proportion of negative links than the HG group (Table S4). Also, from HG to the GRS4 group, the modularity value decreases gradually. The average path length in the GRS4 group also decreased significantly. In contrast, the clustering coefficient and average degree increased for the GRS4 group (Table S4).

Besides, through the interaction network, we obtained the microbe that may be strongly correlated with GRS-related fungus (*Ilyonectria*, *Cylindrocarpon*, and *Fusarium*) mentioned in the previous studies (Table S5) [1, 8, 18, 19]. The results showed that the network relationship of the three fungi was different in the rhizosphere of *P. ginseng* with different degrees of disease, and the interaction relationship may be more in the GRS4 group.

## Soil properties and their relationship with microbial communities

After determining soil physical and chemical properties, we found that the pH of the two severe groups (GRS3 and GRS4) decreased significantly compared with the other three groups (Fig. S4). Significant changes in some nutrients were also observed among the sample groups (Fig. S5). The levels of AP and TP were lower in the two severe groups than in the HG group and mild groups (GRS1 and GRS2), and the AK content of GRS1, GRS3, and GRS4 groups was significantly lower than that of HG and GRS2 groups (Fig. S5D-E and Fig. S5G). Besides, there were no significant changes in other nutrients (OM, TN, AN, and TK). Interestingly, the results on AN differ from previous studies [11]. This may be related to the different study sites and different artificial processing. For rhizosphere enzyme activities, the CAT and PHO enzymes in the GRS3 and GRS4 groups were significantly lower than those in the other three groups (Fig. S6A and Fig. S6D), and the INV in the GRS4 group was also significantly reduced (Fig. S6B).

**Fig. 6:**
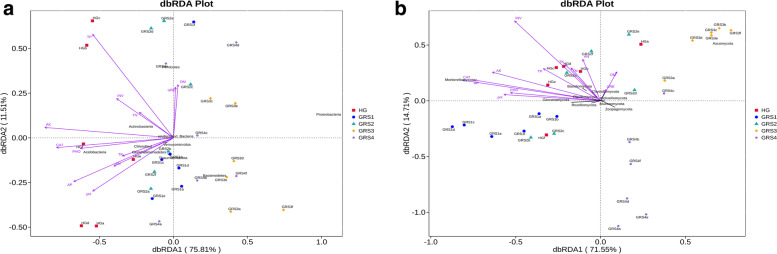
Distance-based redundancy analysis (dbRDA) at the phylum level. (**A**) bacteria; (**B**) fungi. The length of arrows represents the degree of correlation between environmental factors and community distribution and species distribution

The dbRDA results for fungi and bacteria demonstrate the relationship between microorganisms and environmental factors. The envfit function was used to test the significance of each environmental factor, and the results of the significance analysis of the environmental factors are given in Table S6. In Fig. 6 A, axis 1, and axis 2 accounts for 75.81% and 11.51% of the total constrained variance. TP, AK, CAT, AP, PHO, and pH were significantly correlated with bacterial phylum composition. In the fungi plot, axis 1 and axis 2 account for 71.55% and 14.71%, respectively, and INV, CAT, AK, and AP are most closely related to the community composition of fungi (Fig. 6B). Then, the correlation analysis of these key environmental factors with the alpha diversity index, phylum level, and genus level of the bacterial community is shown in Fig. S7. In the results, we found no significant correlation (correlation coefficients were all greater than -0.8 or less than 0.8) in the data. The correlation between these key environmental factors and the fungal community is shown in Fig. S8. The correlation coefficient between *Rhizophagus* and AK and AP was greater than 0.8 (Fig. S8C).

## Discussion

GRS is one of the most concerned *P. ginseng* diseases at present. In recent years, GRS has appeared on a large scale in some *P. ginseng* producing areas in China, seriously damaging the *P. ginseng* industry. As a root disease, a thorough understanding of its rhizosphere characteristics is essential [20]. In this study, to provide a basis for soil improvement and disease control, we made a more detailed division of the disease (from HG to GRS4) to comprehensively reveal the pattern of rhizosphere changes in this *P. ginseng* disease’s background.

## Changes of microbial diversity and richness in rhizosphere

Microbial diversity and balance are critical to plant health [21]. The resistance of the microbial community to pathogen invasion is related to its diversity level [22]. Through the statistics of the Shannon index and Simpson index of each group, we found that only the bacterial diversity of GRS2 was significantly higher than other groups (Table 1). Also, by comparing the differences in the number of OTUs, observed species index and Chao1 index of each group, we found that the richness of the rhizosphere bacterial community in the GRS2 group may be significantly higher in the HG group, while the richness of the rhizosphere bacterial community in the GRS3 group is not significantly different from that in the HG group (Table 1). This result seems to imply that GRS2 (red skin area 25 to 50%) is a grade of concern because of the turning point of changes in the *P. ginseng* rhizosphere’s bacterial diversity. The bacterial community richness also changed significantly from the GRS2 grade.

For fungi, the Shannon index and Simpson index were significantly lower in the severe disease groups (GRS3 and GRS4) than in the HG group. There were no significant changes among the groups except for significant differences in the observed species index and Chao1 index between GRS2 and GRS3 (Table 1). This result indicates that the diseased *P. ginseng* rhizosphere (especially in the severe groups) was affected, while the richness was largely unaffected. It can be seen that the balance of microorganisms changed in the rhizosphere of disease ginseng, which may be related to GRS to some extent.

### Changes of microbial community composition in rhizosphere

Soil microbial community composition directly or indirectly affects soil physical and chemical properties, soil C/N cycle, and plant resistance to external stress [23–25]. Therefore, the analysis of soil community composition is essential. To better reflect the nonlinear structure of the ecological data and the inter- and intra-group differences in the samples, we performed ANOSIM analysis and NMDS analysis based on OTUs level (Fig. 1, Table S3). The bacteria in HG and GRS1 groups were separated from the other three groups, which means that the bacteria in the rhizosphere had a continuous change process with the development of the disease. At the later stage of development (from GRS2 to GRS4), the bacterial community composition among the groups was more similar. By further UPGMA clustering analysis, we also found that the bacterial HG group samples were isolated clusters. Simultaneously, there was no obvious clustering among other diseased groups, which confirmed the similarity of bacterial communities in the rhizosphere of *P. ginseng* with different diseased degrees (Fig. 2 A).

For the ANOSIM analysis and NMDS analysis of fungi, we still found that the HG group was significantly separated from the GRS3 group and the GRS4 group. Interestingly, the groups were not separated by the degree of disease development (Fig. 1B, Table S3). The possible reason is that the fungal community develops in fluctuation rather than gradual as the disease progresses. In terms of fungi, in addition to the HG group, the GRS3 group and GRS4 group also had obvious clustering, which seemed to indicate that the fungal microbial community in the severe groups again formed a stable state different from the HG group (Fig. 2B). Based on the above, it was found that there were differences in microbial community composition in the rhizosphere of *P. ginseng* with different disease degrees. Besides, in the rhizosphere of severe groups (GRS3 and GRS4), the fungal community may be more stable than the bacterial community. GRS is a typical soil-borne disease, and the changes of bacterial and fungal community composition in soil always test the defense function of ginseng. Combined with the analysis of the experimental results, the composition of soil microorganisms of diseased ginseng has significantly changed, and these changes seem to be developing in a direction that is not conducive to the growth of ginseng.

### Characteristics of microbial community taxa variation in rhizosphere

Determining the main microbial communities and searching for different communities in the soil is an important measure to explore the causes of GRS. Proteobacteria is the most dominant phylum among bacteria, consistent with the previous study [26]. Its abundance in the rhizosphere of diseased *P. ginseng* is higher than that of the HG group (Fig. 2 A and Fig. S2). The increase of Bacteroidetes abundance in GRS3 and GRS4 groups (Fig. S2) may be due to its good degradation ability, which is more suitable for survival in the fiercely competitive soil [27]. The abundance of Actinobacteria, Chloroflexi, and Gemmatimonadetes was reduced in severe groups (GRS3 and GRS4) (Fig. S2). Actinobacteria contribute to the decomposition of OM [28], but the ecological functions of Chloroflexi and Gemmatimonadetes were unclear. In the LEfSe analysis, 24 biomarkers were obtained and distributed in four groups (Fig. 3 A). We also identified Acidobacteria as a potential biomarker for the HG group, which has been reported to degrade plant-derived OM specifically and is more abundant in soils where more plants grow [29]. In the GRS1 group, Rhizobiales, a clade of Alphaproteobacteria, were screened out and reported to fix nitrogen or as a pathogen [30]. Most biomarkers in the GRS3 group belongs to Proteobacteria, and the others belong to Bacteroidetes. Rhizobiaceae is a biomarker of the GRS4 group. Although it has been reported that it can cause plant diseases, it is unclear whether it is related to GRS [31].

The fungal community’s dominant phyla are Ascomycota, Mortierellomycota, and Basidiomycota (Fig. 2B). In severe groups, the dominance of Mortierellomycota decreased (Fig. S3). Besides, there are Glomeromycota and Mucoromycota that decrease in abundance as the disease progresses. In the present study, we still found changes in the abundance of *Ilyonectria* in different groups at the genus level. At the same time, at the species level, the abundance of *Ilyonectria robusta* in the rhizosphere soil of *P. ginseng* increased with the increase of the disease degree *Ilyonectria coprosmae* was also high in the severe groups (Fig. S3). We also found the fungi genus (*Trichosporon* and *Paraglomus*), whose abundance decreases with disease development (Fig. S3). Moreover, *Cylindrocarpon* were found to have a high abundance in the GRS4 group. Besides, we obtained specific biomarkers for each group through LEfSe analysis, such as Glomeromycota, *Fusarium*, and *Chaetomium* of the HG group (Fig. 3 C). In the GRS3 group, biomarkers all belong to Ascomycota, the soil ecosystem’s main fungal decomposer (Fig. 3D) [32, 33]. Combined with the results of previous studies, it was speculated that the abundance of *P. ginseng* rhizosphere *Cylindrocarpon* and *Ilyonectria* (especially *Ilyonectria robusta*) might be closely related to the occurrence of cultivated red skin. Other biomarkers except *Ilyonectria* and *Cylindrocarpon* have not been reported to be associated with GRS, although these biomarkers are associated with plant diseases [34, 35].

In general, we used statistical methods to look for changes in the composition of rhizosphere microbial communities with different disease degrees. Although the variation characteristics of the abundance of some taxa in the rhizosphere at different disease degrees have been found, their ecological function is still unclear. But the way to a screening of different communities in soil microorganisms, especially the screening of biomarkers, provides a microbial solution strategy for using the current research results to control GRS and provides a reference for the artificial synthesis of soil microbial fertilizer. Biologicals containing beneficial bacteria have already contributed to the treatment of pests and diseases of other plants [36, 37].

### Changes of the microbial interactions in the networks

Fluctuating or unstable communities commonly lead to outbreaks of disease [38]. Co-occurrence networks can be constructed to analyze the interaction and co-existence patterns among different microorganisms, which is crucial to our further understanding of the changes in the rhizosphere with GRS development [26].

Figure 4 shows that the keystone phylum has not changed in the *P. ginseng* rhizosphere with the disease’s development. However, it should be noted that Proteobacteria accounts for a larger proportion in GRS3 and GRS4 group. Proteobacteria could exploit labile carbon sources and produce extracellular polysaccharides to bind sand particles, and this may mean that the severe groups’ rhizosphere has a stronger nutrient metabolism [39, 40]. Further, we found that links and average degree were fluctuating (from HG to GRS4). The average path length of the GRS4 group was significantly lower than the other four groups (Table S4). Thus, with the disease’s development, the stability and complexity of the rhizosphere bacterial community of *P. ginseng* underwent multiple changes. Also, by counting the ratio of positive to negative links, we can predict that there may be more interspecific competition and ecological niche separation in the GRS groups than in the HG group (Table S4) [36].

For the fungal networks, we can find that the keystone phylum of the rhizosphere was Ascomycota, unchanged at all disease development stages. It can be seen that in *P. ginseng* rhizosphere, Ascomycota was essential for resisting the harsh environment and maintaining system stability [41]. Through the statistics of topology parameters, we found that from HG to GRS4, the number of links, clustering coefficient, and the average degree were the lowest in the GRS1 group and then gradually increased (Table S4). It can be seen that in the early stage (GRS1) of *P. ginseng* disease, the interaction of the fungal community decreases. Simultaneously, from the change of the proportion of positive and negative correlation connections, the competitive relationship among fungal species in the GRS1 group may have decreased sharply (Table S4). In the rhizosphere of the GRS4 group, a more complex fungal community may have formed again. In general, the competitive relationships of fungi in diseased *P. ginseng* rhizosphere were all smaller than those in the HG rhizosphere. Also, the trends of positive correlations between bacterial and fungal networks in the rhizosphere of *P. ginseng* with different degrees of disease and the changes of the proportion of negative correlations were different, probably because bacteria generally grow faster than fungi and intensify the competition for nutrients. Another possible reason is that fungi and bacteria respond differently to the secretions of diseased *P. ginseng* roots.

In previous studies, GRS was closely related to fungi [8, 42]. Based on the interaction network, we identified microorganisms that interacted closely with the reported potentially pathogenic fungi. *Simplicillium*, which is extremely negatively correlated with *Ilyonectria*, is reported as a biological control agent [43]. Although these microorganisms, such as *Musicillium* and *Arachnotheca*, have not been fully studied, establishing the networks also provide a basis for the future search for microorganisms that have antagonistic interactions with pathogens, thus, for biocontrol development. The establishment of co-occurrence networks further provides interaction between communities in the soil, and antagonistic or symbiotic relationships can be utilized for biological control. Especially after screening out the different microbial communities, the community relationship in GRS soil is clearer, which provides the possibility for the targeted search and synthesis of biological agents.

### Changes of physicochemical properties and their relationship to microbial communities

Soil physicochemical properties are closely related to plant diseases and reflect the adaptability of plants to a certain extent. And, changes in the soil’s physicochemical properties directly determine the structural composition of the microbial community [44, 45]. In this study, the rhizosphere’s pH, nutrient composition, and enzyme activity were measured from HG to the GRS4 group. From the GRS2 group, the rhizosphere pH decreased significantly (Fig. S4). Compared with other groups, AP and TP in the rhizosphere of the severe groups (GRS3 and GRS4) were reduced considerably. Also, AK’s content was different among the groups, while there was no significant difference in other nutrients (Fig. S5). Besides, the activities of three enzymes (CAT, INV, and PHO) were significantly decreased in the rhizosphere of the severe groups (GRS3 and/or GRS4), while the activity of the URE was not significantly changed (Fig. S6). The causes of the changes mentioned above in physicochemical properties may be related to GRS occurrence that affects the production of enzymes and the conversion of nutrients in the soil or increase nutrient consumption [46, 47]. It is also associated with the release and accumulation of secretions from the root of diseased *P. ginseng* [3]. Simultaneously, the decrease of pH, nutrient composition and enzyme activity in the rhizosphere of *P. ginseng* will affect the disease resistance of *P. ginseng* itself and increase the morbidity.

To explore the microbial-environmental linkages and gain insight into the key factors influencing microbial communities’ changes, we used dbRDA to examine microbial communities and environmental factors [48, 49]. In the dbRDA results for bacteria, TP, AK, CAT, PHO, and AP were the main factors determining the distribution of bacterial community composition and taxa changes (Fig. 6 A). In the dbRDA analysis of fungi, INV, CAT, AK and AP were significant factors affecting the structure change of the fungal community (Fig. 6B). Furthermore, we further analyzed the correlation between these key environmental factors and microbial communities. This study suggests that these environmental factors may focus on future research on soil improvement due to their close correlation with bacterial and fungal communities.

## Conclusions

We studied the changes in the soil ecological environment of *P. ginseng* rhizosphere with different red skin degree in the same *P. ginseng* farm. With the red skin degree development, the bacterial community’s diversity first increased and then decreased, and the richness also fluctuated. The fungal community’s diversity decreased obviously during the severe disease, but the richness was not affected. The rhizosphere of *P. ginseng* at different stages showed obvious differences, especially between HG and severe groups (GRS3 and GRS4). Besides, we also explored the changes of microbial taxa in different classification levels from HG to GRS4, and potential biomarkers for different groups. We established the interaction networks of rhizosphere microorganisms under this disease’s background, and the keystone phylum of different groups of bacteria and fungi remained unchanged, namely Proteobacteria and Ascomycota. As the disease progressed, the bacterial community underwent multiple changes from complex and stable to simple and unstable. The GRS group also had more inter-competition and ecological niche segregation than the HG group. The fungal community’s stability decreased significantly in the early stages of the disease, followed by forming a stable and complex fungal community. In contrast to bacteria, the GRS groups significantly increased interspecies cooperation and ecological niche overlap in the fungal network than the HG group. In the network, we also obtained several microorganisms closely related to the potential pathogenic fungi, which can provide the basis for biological control. Soil TP, AK, AP, CAT, INV are the key factors influencing the microbial communities. Overall, our study comprehensively analyzed the variation characteristics of the *P. ginseng* rhizosphere, providing evidence for disease control and soil improvement.

## Materials and methods

### Site description and soil sample collection

In the previous investigation, the *P. ginseng* grown at a *P. ginseng* farm showed more red skin root in Hunchun city (42.86′N and 130.37′E) in northeast China [50]. This area has a temperate oceanic monsoon climate. The *P. ginseng* farm had an average annual rainfall of 757 mm and an average yearly temperature of 3.5 °C during *P. ginseng* cultivation (2015 to 2019). Furthermore, this farm is the first to grow *P. ginseng*, the application of pesticides strictly complies with the “Ginseng safe production technical specification of pesticide application (DB22/T 1233-2019)”. Pesticides regularly applied, including carbendazim, tiandashenbao, and metalaxyl, in *P. ginseng* cultivation.

The sampling date is July 2020. All soil samples were collected from the above *P. ginseng* farm with *P. ginseng* (5-year-old). The rhizosphere soil was divided into five grades according to the degree of disease of *P. ginseng*, namely: healthy *P. ginseng* (HG), rust root area greater than 0, less than or equal to 25% (GRS1); rust root area greater than 25%, less than or equal to 50% (GRS2); rust root area greater than 50%, less than or equal to 75% (GRS3); and rust root area greater than 75% (GRS4). Here, the rust root area statistics include taproots and fibrous roots. Six rhizosphere soil samples were collected at each grade, a total of 30 samples (30 *P. ginseng* plants). The rhizosphere soil was collected according to Riley and Barber’s standards [51, 52]. In brief, dig out the complete *P. ginseng* roots from the soil, gently shake off the large blocks, and then obtain the *P. ginseng* root’s rhizosphere. Each soil sample was sieved through a 2 mm plastic mesh. Then, one part of each sample was stored at -80℃ for DNA extraction, and the other part was air-dried at ambient temperature for determination of pH, enzyme activity, and soil nutrients.

### DNA extraction, PCR amplification, and Illumina NovaSeq sequencing

Total genome DNA from samples was extracted using the CTAB method [53, 54]. DNA concentration and purity were monitored on 1% agarose gels. According to the concentration, DNA was diluted to 1 ng/µL using sterile water.

The amplicon generation refers to the experimental method of Wang et al. [55]. 16 S rRNA genes of V3-V4 regions, and ITS genes of ITS1-1 F regions were amplified used specific primer 341 F(5´-CCTACGGGNGGCWGCAG-3′)-806R (5´-GGACTACHVGGGTWTCTAAT-3′) [56] and ITS1F (5´-CTTGGTCATTTAGAGGAAGTAA-3′)-ITS1R(5´-GCTGCGTTCTTCATCGATGC-3′) [57] with the barcode, respectively. All PCR reactions were carried out with 15 µL of Phusion® High-Fidelity PCR Master Mix (New England Biolabs), 2 µM of forward and reverse primers, and about 10 ng template DNA. Thermal cycling consisted of initial denaturation at 98℃ for 1 min, followed by 30 cycles of denaturation at 98℃ for 10 s, annealing at 50℃ for 30 s, and elongation at 72℃ for 30 s. Finally, the temperature was maintained at 72℃ for 5 min.

Mix the same volume of 1X loading buffer (contained SYB green) with PCR products and operate electrophoresis on 2% agarose gel for detection. PCR products were mixed in equidensity ratios. Then, the mixture of PCR products was purified with the Qiagen Gel Extraction Kit (Qiagen, Germany).

Sequencing libraries were generated using TruSeq® DNA PCR-Free Sample Preparation Kit (Illumina, USA) following the manufacturer’s recommendations, and index codes were added. The library quality was assessed on the Qubit@ 2.0 Fluorometer (Thermo Scientific) and Agilent Bioanalyzer 2100 system. At last, the library was sequenced on an Illumina NovaSeq6000 platform, and 250 bp paired-end reads were generated at Novogene Bioinformatics Technology Co., Ltd. (Beijing, China). The raw reads were deposited into the National Center for Biotechnology Information database (Accession Number: PRJNA704771 and PRJNA704998).

### Data analysis

Paired-end reads were assigned to samples based on their unique barcode and truncated by cutting off the barcode and primer sequence. Paired-end reads were merged using FLASH (V1.2.7, http://ccb.jhu.edu/software/FLASH/), a high-speed and accurate analysis tool, which was designed to merge paired-end reads when at least some of the reads overlap the read generated from the opposite end of the same DNA fragment, and the splicing sequences were called raw tags [58]. Quality filtering on the raw tags was performed under specific filtering conditions to obtain the high-quality clean tags according to the QIIME (V1.9.1, http://qiime.org/scripts/split_libraries_fastq.html) quality-controlled process [59, 60]. The tags were compared with the reference database (Silva database, using UCHIME algorithm (UCHIME Algorithm, http://www.drive5.com/usearch/manual/uchime_algo.html) to detect chimera sequences, and then the chimera sequences were removed [61, 62]. Then the effective tags were finally obtained. Detailed quality control information is shown in Table S1 and Table S2.

Sequences analysis was performed by Uparse software (Uparse v7.0.1001, http://drive5.com/uparse/) [63], OTUs were clustered with 97% identity, and then species annotation was performed on the OTUs sequences. Furthermore, all samples’ rarefaction curves also indicate the reliability of the sampling depth (Fig. S1). The representative sequence for each OTU was screened for further annotation. For each representative sequence, the Silva Database (http://www.arb-silva.de/) was used based on the Mothur algorithm to annotate taxonomic information [64]. To study the phylogenetic relationship of different OTUs, and the difference of the dominant species in different samples (groups), multiple sequence alignment was conducted using the MUSCLE software (Version 3.8.31, http://www.drive5.com/muscle/) [65]. OTUs abundance information was normalized using a standard of sequence number corresponding to the sample with the least sequences. Subsequent analysis of alpha diversity and beta diversity were all performed basing on this output normalized data.

### Rhizosphere pH, nutrients, and enzyme activity

The rhizosphere’s pH value was measured with a pH meter/potentiometer under the soil: water ratio of 1:2.5. The soil organic matter (OM) content was measured by the potassium dichromate external heating method. The total N (TN) was determined by the Kjeldahl method; the TP was determined by the alkali fusion molybdenum antimony colorimetry (China HJ 632-2011); the total K (TK) was determined by the sodium hydroxide fusion flame photometric method. The AP was determined by NaHCO_3_ extraction molybdenum-antimony colorimetry; the available N (AN) was determined by the alkali diffusion method; AK was determined with the ammonium acetate extraction fame photometric method [66].

The activities of CAT, invertase (INV), urease (URE), and phosphatase (PHO) in the soil of *P. ginseng* were measured. The activity of CAT was determined by KMnO_4_ titration; soil INV activity was determined by 3,5-dinitrosalicylic acid colorimetry; URE activity was determined by indophenol blue colorimetry; PHO activity was determined by disodium phenyl phosphate colorimetry method [67].

### Statistical analysis

Alpha diversity was used to analyze the microbial community diversity in the sample and assess the microbial community’s species richness and diversity differences. Five indices were selected to identify microbial alpha diversity: the observed OTUs number, the observed species number, the Chao1 index, the Simpson index, and the Shannon index [55]. Beta diversity was used for comparative analysis of microbial community composition in different samples. Cluster analysis was preceded by NMDS analysis based on Bray-Curtis distance was performed to reflect inter-group and intra-group relationships. In addition, to determine whether the differences between groups were significantly greater than the differences within groups, ANOSIM analysis was performed in the R software using the ANOSIM function of the vegan package [68]. UPGMA clustering was performed as a hierarchical clustering method to interpret the distance matrix. The Kruskal-Wallis rank-sum test was used to analyze differences in alpha diversity and beta diversity among multiple groups. All of the indices in our samples were calculated with QIIME and displayed with R software.

Metastat analysis was used to carry out permutation tests among groups to study the taxa with significant differences among groups in R software, and False Discovery Rate (FDR)-corrected P value (Q value) <0.05 was considered statistically significant. Besides, to find statistically different biomarkers, the LEfSe analysis was used to identify the differentially abundant taxa. LDA score greater than 4 was used as a potential biomarker (taxonomic level from phylum to species).

Spearman correlation analysis was used to study the mutual change relationship between environmental factors and microbial taxa, and P < 0.05 was considered a significant difference [69]. Furthermore, dbRDA analysis was used to reflect the relationship between community structures and environmental factors [70]. Both analyses were performed using the vegan package in R software.

We calculated the Spearman correlation coefficient for all samples to explore the microbial taxa with close interaction to obtain the taxa correlation coefficient matrix. Then, the co-occurrence network was obtained through filtering. The filtering conditions were as follows: (1) remove connections with a correlation coefficient of less than 0.6 and a P-value greater than 0.05, (2) filter out the self-connection of nodes, and (3) remove connections with node abundance less than 0.005%. To assess non-random patterns in the resulting network, we compared our network against its randomized version (Erdös-Réyni model) using the random networks plugin in Cytoscape (3.8.2) [71–73].

Environmental factor data were analyzed using the SPSS software (IBM Corporation, Armonk, NY, USA), and the results were expressed as the arithmetic mean value ± standard deviation. The differences in the means were compared by the one-way ANOVA test, and post hoc analysis were performed using the Tukey test. Differences at the P < 0.05 level were considered to be statistically significant.

## Supplementary Information

**Additional file 1.**

## Data Availability

Amplicon sequences are available at the NCBI database (Accession Number: PRJNA704771 and PRJNA704998). The OTUs sequences can be obtained from the corresponding author.
