# Supplementary material for: Rhizosphere analysis of field-grown Panax ginseng with different degrees of red skin provides the basis for preventing red skin syndrome
Source: BMC Microbiol. 2022 Jan 6;22:12. doi: 10.1186/s12866-021-02430-9 (PMC8734182; doi:10.1186/s12866-021-02430-9)
Supplement: Supplementary file 1 — Additional file 1. [file 12866_2021_2430_MOESM1_ESM.docx]

**Rhizosphere analysis of field-grown *Panax ginseng* with different degrees of red skin provides the basis for preventing red skin syndrome**

Ling Dong^ab^, Xingbo Bian^ab^, Yan Zhao^b^, He Yang^ab^, Yonghua Xu^ab*^, Yongzhong Han^c^ and Lianxue Zhang^ab*^

1. *National& Local Joint Engineering Research Center for Ginseng Breeding and Development, Jilin Agricultural University, Changchun, China*
2. *College of Chinese Medicinal Materials,* *Jilin Agricultural University, Changchun, China*
3. *Jilin Provincial Ginseng and Pilose Antler Office, Changchun, China*

* Corresponding author at: College of Chinese Medicinal Materials, Jilin Agricultural University, Changchun 130118, Jilin Province, China. Tel/Fax: +86 431 84533358, E-Mail address: [xuyonghua777@yeah.net](mailto:xuyonghua777@yeah.net) (Y. Xu), zlx863@163.com (LX. Zhang).

| Sample Name | Raw PE | Raw Tags | Clean Tags | Effective Tags | Q20% | Q30% | GC% | Effective% |
| --- | --- | --- | --- | --- | --- | --- | --- | --- |
| HGa | 85,615 | 81,029 | 78,710 | 62,579 | 98.25 | 94.35 | 56.36 | 73.09 |
| HGb | 96,265 | 85,042 | 80,998 | 63,751 | 97.86 | 93.24 | 55.89 | 66.22 |
| HGc | 80,408 | 65,779 | 61,796 | 47,380 | 97.78 | 93.04 | 55.65 | 58.92 |
| HGd | 98,106 | 90,807 | 87,457 | 61,730 | 98.08 | 93.79 | 56.17 | 62.92 |
| HGe | 79,888 | 65,536 | 61,563 | 48,228 | 97.76 | 93 | 55.73 | 60.37 |
| HGf | 94,140 | 78,934 | 74,625 | 58,806 | 97.9 | 93.29 | 55.92 | 62.47 |
| GRS1a | 92,042 | 87,960 | 86,029 | 63,497 | 98.47 | 94.84 | 55.85 | 68.99 |
| GRS1b | 92,500 | 79,467 | 75,747 | 59,329 | 97.98 | 93.52 | 55.81 | 64.14 |
| GRS1c | 94,033 | 80,050 | 76,054 | 60,210 | 97.88 | 93.28 | 55.6 | 64.03 |
| GRS1d | 85,364 | 72,908 | 69,087 | 53,312 | 97.69 | 92.7 | 55.45 | 62.45 |
| GRS1e | 93,471 | 79,651 | 75,922 | 59,793 | 97.83 | 93.01 | 55.72 | 63.97 |
| GRS1f | 74,375 | 63,201 | 60,130 | 46,846 | 97.8 | 93.05 | 55.48 | 62.99 |
| GRS2a | 90,189 | 85,752 | 83,177 | 64,929 | 98.25 | 94.32 | 55.89 | 71.99 |
| GRS2b | 84,354 | 71,589 | 67,421 | 52,960 | 97.73 | 92.89 | 55.7 | 62.78 |
| GRS2c | 97,021 | 85,077 | 81,859 | 66,295 | 98.24 | 94.11 | 55.12 | 68.33 |
| GRS2d | 89,102 | 77,359 | 73,646 | 56,845 | 97.82 | 93.2 | 55.55 | 63.8 |
| GRS2e | 91,689 | 75,792 | 71,497 | 56,277 | 97.79 | 92.97 | 55.33 | 61.38 |
| GRS2f | 91,961 | 80,366 | 76,214 | 58,618 | 97.8 | 93.08 | 55.69 | 63.74 |
| GRS3a | 83,697 | 80,775 | 78,922 | 63,764 | 98.56 | 95.15 | 55.48 | 76.18 |
| GRS3b | 98,751 | 83,136 | 78,603 | 63,378 | 97.99 | 93.58 | 55.1 | 64.18 |
| GRS3c | 85,830 | 72,948 | 69,249 | 54,929 | 97.89 | 93.25 | 55 | 64 |
| GRS3d | 93,352 | 79,788 | 75,556 | 58,799 | 97.91 | 93.37 | 55.16 | 62.99 |
| GRS3e | 92,548 | 78,912 | 74,609 | 58,995 | 97.93 | 93.36 | 55.09 | 63.75 |
| GRS3f | 81,304 | 68,987 | 65,648 | 51,682 | 97.97 | 93.39 | 54.23 | 63.57 |
| GRS4a | 80,847 | 77,470 | 75,693 | 59,487 | 98.46 | 94.83 | 55.6 | 73.58 |
| GRS4b | 89,386 | 75,687 | 71,890 | 57,109 | 97.88 | 93.29 | 55.39 | 63.89 |
| GRS4c | 88,601 | 73,639 | 69,639 | 53,881 | 97.93 | 93.39 | 55.49 | 60.81 |
| GRS4d | 87,799 | 76,669 | 73,273 | 58,853 | 98.14 | 93.82 | 54.87 | 67.03 |
| GRS4e | 90,104 | 76,512 | 72,220 | 56,546 | 97.8 | 93.07 | 55.68 | 62.76 |
| GRS4f | 87,081 | 76,750 | 73,711 | 59,349 | 98.18 | 94.01 | 54.47 | 68.15 |

**Table S1.** Statistical results of bacterial sequencing data processing. Raw PE represents original PE reads; Raw Tags refers to the sequence of Tags that have been stitched together; Clean Tags refers to the sequence of Tags after filtering out low quality and short lengths; Effective Tags refer to the sequence of Tags filtered through chimeras and eventually used for subsequent analysis.Q20 and Q30 are the percentage of the Effective Tags with base quality values greater than 20(sequencing error rate less than 1%) and 30(sequencing error rate less than 0.1%).GC (%) represents the amount of GC bases in Effective Tags; Effective (%) indicates the percentage of the number of Effective Tags and the number of Raw PE.

| Sample Name | Raw PE | Raw Tags | Clean Tags | Effective Tags | Q20% | Q30% | GC% | Effective% |
| --- | --- | --- | --- | --- | --- | --- | --- | --- |
| HGa | 85,588 | 84,256 | 83,598 | 66,365 | 98.5 | 95.73 | 48.01 | 77.54 |
| HGb | 99,861 | 96,247 | 94,317 | 66,230 | 98.48 | 95.91 | 46.92 | 66.32 |
| HGc | 88,507 | 86,344 | 84,406 | 66,519 | 98.52 | 96.03 | 46.48 | 75.16 |
| HGd | 91,708 | 88,589 | 86,730 | 64,808 | 98.54 | 96.1 | 46.76 | 70.67 |
| HGe | 97,422 | 95,019 | 93,557 | 65,882 | 98.64 | 96.31 | 46.55 | 67.63 |
| HGf | 81,911 | 79,550 | 77,903 | 65,961 | 98.48 | 95.9 | 46.33 | 80.53 |
| GRS1a | 87,394 | 86,206 | 85,590 | 62,195 | 98.54 | 95.81 | 45.87 | 71.17 |
| GRS1b | 96,387 | 93,800 | 92,038 | 66,847 | 98.58 | 96.21 | 46.59 | 69.35 |
| GRS1c | 87,016 | 83,677 | 82,073 | 69,512 | 98.6 | 96.21 | 45.08 | 79.88 |
| GRS1d | 92,379 | 89,030 | 87,239 | 63,725 | 98.59 | 96.19 | 44.78 | 68.98 |
| GRS1e | 87,657 | 85,047 | 83,652 | 62,388 | 98.66 | 96.37 | 45.41 | 71.17 |
| GRS1f | 80,173 | 76,862 | 74,893 | 62,828 | 98.5 | 95.94 | 45.89 | 78.37 |
| GRS2a | 90,994 | 89,787 | 89,284 | 64,704 | 98.67 | 96.08 | 46.13 | 71.11 |
| GRS2b | 92,755 | 90,133 | 87,717 | 62,793 | 98.46 | 95.9 | 45.75 | 67.7 |
| GRS2c | 87,390 | 84,680 | 82,680 | 65,294 | 98.5 | 95.97 | 45.78 | 74.72 |
| GRS2d | 99,856 | 96,210 | 94,492 | 63,276 | 98.56 | 96.07 | 47.26 | 63.37 |
| GRS2e | 90,427 | 88,354 | 86,468 | 65,837 | 98.61 | 96.3 | 47.24 | 72.81 |
| GRS2f | 91,310 | 88,178 | 86,752 | 60,319 | 98.58 | 96.14 | 46.75 | 66.06 |
| GRS3a | 93,008 | 91,856 | 91,234 | 61,251 | 98.73 | 96.17 | 46.26 | 65.86 |
| GRS3b | 98,092 | 96,009 | 94,382 | 66,927 | 98.61 | 96.2 | 47.7 | 68.23 |
| GRS3c | 80,456 | 78,219 | 76,725 | 65,990 | 98.64 | 96.22 | 48.04 | 82.02 |
| GRS3d | 85,283 | 82,485 | 80,804 | 68,095 | 98.64 | 96.31 | 48 | 79.85 |
| GRS3e | 89,533 | 86,615 | 85,178 | 62,403 | 98.7 | 96.36 | 48.03 | 69.7 |
| GRS3f | 78,007 | 75,630 | 74,537 | 67,623 | 98.8 | 96.54 | 47.88 | 86.69 |
| GRS4a | 92,888 | 91,875 | 91,267 | 62,682 | 98.78 | 96.38 | 46.73 | 67.48 |
| GRS4b | 90,815 | 88,296 | 86,888 | 61,518 | 98.72 | 96.51 | 47.56 | 67.74 |
| GRS4c | 80,076 | 77,631 | 75,862 | 68,017 | 98.59 | 96.13 | 47.93 | 84.94 |
| GRS4d | 94,494 | 91,729 | 89,907 | 61,778 | 98.67 | 96.39 | 46.87 | 65.38 |
| GRS4e | 85,089 | 81,690 | 80,047 | 68,647 | 98.36 | 95.53 | 47.92 | 80.68 |
| GRS4f | 88,486 | 87,507 | 85,527 | 66,565 | 98.67 | 96.45 | 46.11 | 75.23 |

**Table S2.** Statistical results of fungal sequencing data processing. Raw PE represents original PE reads; Raw Tags refers to the sequence of Tags that have been stitched together; Clean Tags refers to the sequence of Tags after filtering out low quality and short lengths; Effective Tags refer to the sequence of Tags filtered through chimeras and eventually used for subsequent analysis. Q20 and Q30 are the percentage of the Effective Tags with base quality values greater than 20(sequencing error rate less than 1%) and 30(sequencing error rate less than 0.1%). GC (%) represents the amount of GC bases in Effective Tags; Effective (%) indicates the percentage of the number of Effective Tags and the number of Raw PE.

| **bacteria** |  |  |  | **Fungi** |  |  |
| --- | --- | --- | --- | --- | --- | --- |
| Group | R-value | P-value |  | Group | R-value | P-value |
| HG-GRS1 | 0.9185 | 0.003 |  | HG-GRS1 | 0.9685 | 0.001 |
| HG-GRS2 | 0.9407 | 0.004 |  | HG-GRS2 | 0.9769 | 0.002 |
| HG-GRS3 | 1 | 0.004 |  | HG-GRS3 | 1 | 0.003 |
| HG-GRS4 | 0.8278 | 0.005 |  | HG-GRS4 | 0.9204 | 0.005 |
| GRS1-GRS2 | 0.5463 | 0.002 |  | GRS1-GRS2 | 0.7593 | 0.001 |
| GRS1-GRS3 | 0.963 | 0.004 |  | GRS1-GRS3 | 0.9833 | 0.003 |
| GRS1-GRS4 | 0.4648 | 0.001 |  | GRS1-GRS4 | 0.9741 | 0.003 |
| GRS2-GRS3 | 0.9611 | 0.005 |  | GRS2-GRS3 | 0.9648 | 0.001 |
| GRS2-GRS4 | 0.09537 | 0.16 |  | GRS2-GRS4 | 0.9204 | 0.004 |
| GRS3-GRS4 | 0.7333 | 0.003 |  | GRS3-GRS4 | 0.9907 | 0.003 |

**Table S3.** ANOSIM analysis results. HG (healthy ginseng), GRS1 (rust root area greater than 0, less than or equal to 25%), GRS2 (red skin root area greater than 25%, less than or equal to 50%), GRS3 (rust root area greater than 50%, less than or equal to 75%) and GRS4 (rust root area greater than 75%).

|  | Nodes | Total link | Positive links (percentage) | Negative links (percentage) | Modularity | CC | AD | APL |  | Modularity | CC | APL |
| --- | --- | --- | --- | --- | --- | --- | --- | --- | --- | --- | --- | --- |
| **Bacteria**  Non-Random network |  |  |  |  |  |  |  |  | Random networks |  |  |  |
| HG | 289 | 3248 | 2073 (63.8%) | 1175 (36.2%) | 0.5663422 | 0.4992137 | 18.76125 | 4.941463 |  | 0.227 | 0.079 | 2.081 |
| GRS1 | 284 | 2839 | 1659 (58.4%) | 1180 (41.6%) | 0.5335726 | 0.4705993 | 17.99296 | 4.910784 |  | 0.224 | 0.068 | 2.151 |
| GRS2 | 292 | 3227 | 1790 (55.5%) | 1437 (44.5%) | 0.5140345 | 0.5030765 | 20.11644 | 4.806641 |  | 0.226 | 0.075 | 2.096 |
| GRS3 | 284 | 2789 | 1695 (60.8%) | 1094 (39.2%) | 0.5446874 | 0.4699048 | 17.64789 | 5.045968 |  | 0.226 | 0.070 | 2.166 |
| GRS4 | 290 | 3600 | 2136 (59.3%) | 1464 (40.7%) | 0.4940495 | 0.5276348 | 22.82759 | 4.14866 |  | 0.231 | 0.083 | 2.023 |
| **Fungi** |  |  |  |  |  |  |  |  |  |  |  |  |
| HG | 88 | 363 | 234 (64.5%) | 129 (35.5%) | 0.6109209 | 0.5060241 | 6.227273 | 3.195169 |  | 0.228 | 0.080 | 2.325 |
| GRS1 | 86 | 333 | 278 (83.5%) | 55 (16.5%) | 0.5981724 | 0.4609375 | 5.72093 | 2.63789 |  | 0.198 | 0.110 | 2.378 |
| GRS2 | 88 | 347 | 233 (67.1%) | 114 (32.9%) | 0.5863755 | 0.4710425 | 5.863636 | 4.156273 |  | 0.217 | 0.097 | 2.367 |
| GRS3 | 88 | 368 | 265 (72.0%) | 103 (28.0%) | 0.5654925 | 0.5232068 | 6.340909 | 3.437685 |  | 0.221 | 0.088 | 2.308 |
| GRS4 | 87 | 438 | 312 (71.2%) | 126 (28.8%) | 0.4983225 | 0.5516857 | 8.045977 | 2.816084 |  | 0.220 | 0.121 | 2.156 |

**Table S4.** Network topological characteristics of microbial communities. HG (healthy ginseng), GRS1 (rust root area greater than 0, less than or equal to 25%), GRS2 (red skin root area greater than 25%, less than or equal to 50%), GRS3 (rust root area greater than 50%, less than or equal to 75%) and GRS4 (rust root area greater than 75%).

| **HG** |  |  | **GRS4** |  |  |
| --- | --- | --- | --- | --- | --- |
|  |  | SCC |  |  | SCC |
| Ilyonectria | Pleotrichocladium | 0.828571429 | Fusarium | Chrysosporium | 0.828571429 |
| Ilyonectria | Pseudallescheria | -0.81167945 | Fusarium | Pseudogymnoascus | 0.898645105 |
| Ilyonectria | Zasmidium | 0.81167945 | Fusarium | Aspergillus | 0.885714286 |
| Ilyonectria | Colletotrichum | 0.898645105 | Fusarium | Paraglomus | -0.885714286 |
| Cylindrocarpon | Minimedusa | -0.828571429 | Fusarium | Arizonaphlyctis | -0.845154255 |
| Cylindrocarpon | Microdochium | 0.828571429 | Fusarium | Coniochaeta | -0.828571429 |
|  |  |  | Fusarium | Pseudosigmoidea | 0.81167945 |
|  |  |  | Fusarium | Cylindrocarpon | 0.840668002 |
|  |  |  | Ilyonectria | Byssonectria | 0.828078671 |
|  |  |  | Ilyonectria | Simplicillium | -0.927633657 |
|  |  |  | Ilyonectria | Musicillium | -0.927633657 |
|  |  |  | Ilyonectria | Pochonia | -0.880406274 |
|  |  |  | Ilyonectria | Arachnotheca | -0.985610761 |
|  |  |  | Ilyonectria | Sagenomella | -0.869656553 |
|  |  |  | Ilyonectria | Corallomycetella | -0.885714286 |
|  |  |  | Ilyonectria | Trichoderma | -0.828571429 |
|  |  |  | Ilyonectria | Penicillium | -0.942857143 |

**Table S5** Fungus in interaction networks that may be associated with diseases. SCC (Spearman correlation coefficient). HG (healthy ginseng), and GRS4 (rust root area greater than 75%).

| bacteria |  |  |  |  |  | Fungi |  |  |  |  |
| --- | --- | --- | --- | --- | --- | --- | --- | --- | --- | --- |
|  | CAP1 | CAP2 | r2 | Pr (>r) |  |  | CAP1 | CAP2 | r2 | Pr (>r) |
| pH | -0.889 | -0.45791 | 0.305657 | 0.008496 |  | pH | -0.92615 | 0.37715 | 0.24163 | 0.024988 |
| CAT | -0.99222 | -0.12453 | 0.488507 | 0.0005 |  | CAT | -0.89992 | 0.43605 | 0.546583 | 0.0005 |
| INV | -0.9161 | 0.400956 | 0.137439 | 0.126437 |  | INV | -0.71387 | 0.70029 | 0.630943 | 0.00055 |
| URE | 0.13283 | 0.991139 | 0.05359 | 0.47926 |  | URE | -0.03723 | 0.99931 | 0.004813 | 0.938531 |
| PHO | -0.98942 | -0.14508 | 0.312616 | 0.007996 |  | PHO | -0.91833 | 0.39583 | 0.21703 | 0.035482 |
| OM | 0.195045 | 0.980794 | 0.06025 | 0.447776 |  | OM | 0.041627 | 0.99913 | 0.03136 | 0.66067 |
| TN | -0.901356 | 0.433078 | 0.051453 | 0.485757 |  | TN | -0.68632 | 0.727304 | 0.093149 | 0.271864 |
| TP | -0.7422 | 0.670176 | 0.418803 | 0.001499 |  | TP | -0.78583 | 0.61844 | 0.170738 | 0.088956 |
| AN | -0.93079 | -0.36556 | 0.148589 | 0.122439 |  | AN | -0.57766 | 0.81628 | 0.102069 | 0.225887 |
| AK | -0.99995 | -0.00999 | 0.580067 | 0.0005 |  | AK | -0.86473 | 0.50224 | 0.39551 | 0.001 |
| AP | -0.93831 | -0.3458 | 0.406798 | 0.001 |  | AP | -0.89498 | 0.44611 | 0.466004 | 0.001 |
| TK | -0.95438 | -0.2986 | 0.102093 | 0.229385 |  | TK | -0.69837 | 0.71573 | 0.119828 | 0.184408 |

**Table S6.** Significance of environmental factors based on envfit function statistics in dbRDA analysis. CCA1 and CCA2 are cosine values of the angle between environmental factors and the ranking axis; r2 is the coefficient of determination of environmental factors on species distribution; Pr is the significance test of correlation.

**Figure S1.** The rarefaction curves of observed OTUs at OTU level across all samples. A) bacteria; B) fungi.

**Figure S2.** MetaStat analysis of bacterial abundance from phylum level to species level. HG (healthy ginseng), GRS1 (rust root area greater than 0, less than or equal to 25%), GRS2 (red skin root area greater than 25%, less than or equal to 50%), GRS3 (rust root area greater than 50%, less than or equal to 75%) and GRS4 (rust root area greater than 75%).

**Figure S3.** MetaStat analysis of fungi abundance from phylum level to species level. HG (healthy ginseng), GRS1 (rust root area greater than 0, less than or equal to 25%), GRS2 (red skin root area greater than 25%, less than or equal to 50%), GRS3 (rust root area greater than 50%, less than or equal to 75%) and GRS4 (rust root area greater than 75%).

**Figure S4.** PH of rhizosphere soil. Different letters in the same row mean significant difference at P <0.05 among the five treatments. HG (healthy ginseng), GRS1 (rust root area greater than 0, less than or equal to 25%), GRS2 (red skin root area greater than 25%, less than or equal to 50%), GRS3 (rust root area greater than 50%, less than or equal to 75%) and GRS4 (rust root area greater than 75%).

**Figure S5.** Nutrients of rhizosphere soil. Different letters in the same row mean significant difference at P <0.05 among the five treatments. HG (healthy ginseng), GRS1 (rust root area greater than 0, less than or equal to 25%), GRS2 (red skin root area greater than 25%, less than or equal to 50%), GRS3 (rust root area greater than 50%, less than or equal to 75%) and GRS4 (rust root area greater than 75%).

**Figure S6.** Enzyme activities of rhizosphere soil. Different letters in the same row mean significant difference at P <0.05 among the five treatments. HG (healthy ginseng), GRS1 (rust root area greater than 0, less than or equal to 25%), GRS2 (red skin root area greater than 25%, less than or equal to 50%), GRS3 (rust root area greater than 50%, less than or equal to 75%) and GRS4 (rust root area greater than 75%).

**Figure S7.** Correlation analysis of key environmental factors and bacterial community. A) alpha diversity index; B) phylum level; C) genus level.

**Figure S8.** Correlation analysis of key environmental factors and fungal community. A) alpha diversity index; B) phylum level; C) genus level.
